# Gap between national food production and food-based dietary guidance highlights lack of national self-sufficiency

**DOI:** 10.1038/s43016-025-01173-4

**Published:** 2025-05-16

**Authors:** Jonas Stehl, Alexander Vonderschmidt, Sebastian Vollmer, Peter Alexander, Lindsay M. Jaacks

**Affiliations:** 1 Department of Economics, University of Goettingen, Göttingen, Germany; 2 Centre for Modern Indian Studies, University of Goettingen, Göttingen, Germany; 3 Global Academy of Agriculture and Food Systems, The University of Edinburgh, Midlothian, UK; 4 School of Geosciences, The University of Edinburgh, Midlothian, UK

**Keywords:** Economics, Sustainability, Environmental studies

## Abstract

In light of nationalist trends, disruptions to global food supply chains and efforts to concurrently promote sustainable diets, we assess national capacities to achieve dietary guidelines based on domestic production alone. Over a third of all countries cannot meet self-sufficiency for more than two of the seven essential food groups. Low self-sufficiency and overdependence on a few countries for imports threaten their capability to respond to global shocks, particularly for small states.

## Abstract (web summary)

Recent global disruptions have revived the food self-sufficiency debate. This study analyses countries’ trade dependencies and the discrepancy between their domestic food production and guidelines for a healthy and sustainable diet across seven essential food groups.

## Main

Recent disruptions—such as the COVID-19 pandemic^1,2^ and the outbreak of the war in Ukraine^3–5^—have underscored the vulnerability of long food supply chains, prompting renewed discussions on self-sufficiency^6^. In addition, while advocates of the ‘eat local’ movement focus on reducing diet-related emissions, transport contributes only ~5% of food-systems emissions^7,8^.

This raises the question of whether countries can achieve food self-sufficiency. We use Food and Agricultural Organization (FAO) Food Balance Sheets (FBS) 2020 production data and the World Wildlife Fund’s (WWF’s) Livewell diet^9^ to analyse the discrepancy between domestic food production and dietary guidelines across seven food groups.

Previous studies have assessed caloric self-sufficiency at various administrative levels based on current consumption patterns^10,11^. We pursue a more comprehensive approach, focusing on food groups essential for a healthy diet—rather than solely on calories—and analysing both past and future trends in self-sufficiency. Finally, we analyse trade dependencies of countries with low self-sufficiency, emphasizing the critical role of response diversity—the ability of countries to adapt to trade disruptions by diversifying their import sources, as defined by Walker et al.^12^—in building resilient food systems.

**Fig. 1:**
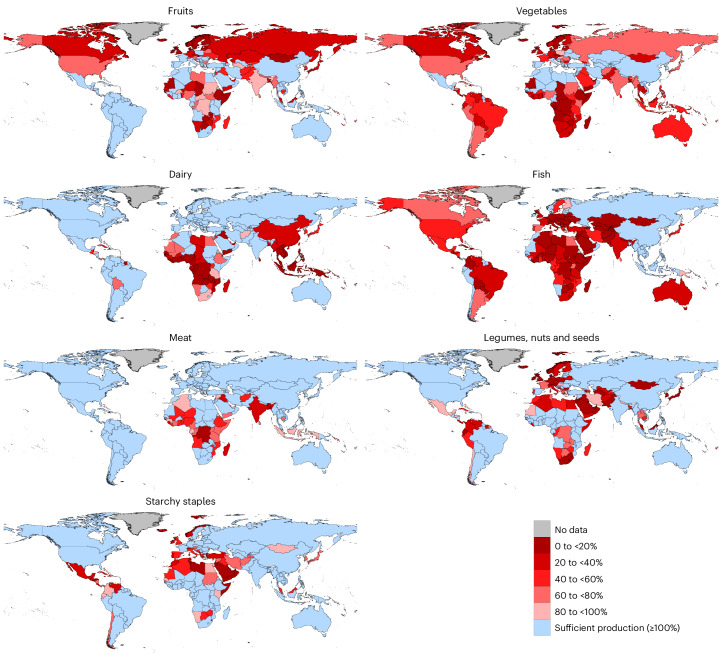
Percentage of self-sufficiency for specific food groups. National food availability from domestic production as proportion from recommended intake by the Livewell diet in grams per capita per day for 187 (dairy, 186) countries in 2020. Thereby, 100% means that all recommendations of a food group are met.

Out of 186 countries, 154 can fulfil the requirements for 2 to 5 out of 7 food groups of the Livewell diet through domestic production (Fig. 1). Only Guyana achieves self-sufficiency for all seven food groups, while China and Vietnam attain six. By contrast, six countries, primarily in the Middle East—Afghanistan, United Arab Emirates, Iraq, China Macao Special Administrative Region, Qatar and Yemen—do not achieve the needs of any food group (Fig. 1). More than one-third of all countries achieve self-sufficiency for two or fewer groups; 25 are in Africa, 10 in the Caribbean, and 7 in Europe. Only one in seven countries achieve self-sufficiency in five or more food groups, most within Europe and South America.

Regarding animal agriculture, meat self-sufficiency is relatively high, with 65% of countries (over)achieving their dietary needs, while sub-Saharan Africa faces considerable deficits. African countries also face challenges in dairy production, along with Oceania; 82% and 83%, respectively, are not able to achieve their dairy needs. Less than half achieve self-sufficiency for dairy (44%), but all European countries can reach their dairy requirements independently. Fish and seafood self-sufficiency is particularly low across most regions, only 25% achieving sufficiency, including Russia and countries in the Pacific region. Globally, 60% of countries cannot cover half of their fish needs (Fig. 1).

Approximately one-half of countries achieve self-sufficiency for starchy staples (45%); legumes, nuts and seeds (46%); and fruits (47%), but fewer than one quarter do so for vegetables (24%). Starchy staple production is insufficient in key regions including Western Asia, the Middle East and North Africa, the Caribbean and Central America, where only Dominica is self-sufficient. Conversely, South America and the Caribbean perform well in fruit production, while all Northern European countries fail to cover even half of their fruit requirements. Vegetable self-sufficiency is high in the Mediterranean and Central Asia, yet 91% of sub-Saharan African countries fall short. Northern Europe, South America and the Caribbean also struggle with vegetable production, with only Guyana achieving sufficiency in these regions (Fig. 1).

**Fig. 2:**
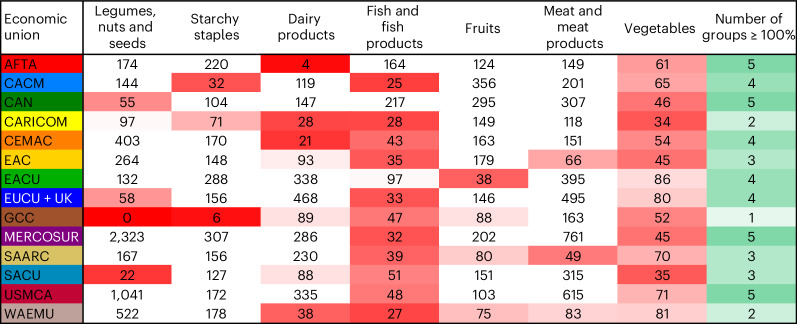
Percentage of self-sufficiency for specific food groups at different regional levels. The numbers are the proportion of domestic production (deducted by food that will not enter the body) of nutritional requirements of the Livewell diet in percentage. Thereby, 100% means the requirements of the respective food group are met. White cells denote full self-sufficiency. Red cells mark a deficit; the darker the red, the larger the gap. Green cells show the number of food groups regions are self-sufficient in; darker green corresponds to a higher count. EUCU + UK, European Union Customs Union and United Kingdom; EACU, Eurasian Customs Union; EAC, East African Community; WAEMU, West African Economic and Monetary Union; SACU, Southern African Customs Union; CEMAC, Communauté Économique et Monétaire de l’Afrique Centrale; MERCOSUR, Mercado Común del Sur; CAN, Andean Community; CARICOM, Caribbean Community; CACM, Central American Common Market; GCC, Gulf Cooperation Council; AFTA, ASEAN Free Trade Area; USMCA, United States–Mexico–Canada Agreement; SAARC, South Asian Association for Regional Cooperation. See Supplementary Fig. 1 and Supplementary Table 1 for further details.

We also examined self-sufficiency within economic unions and the resulting echo patterns observed at the country level. For instance, the Gulf Cooperation Council is self-sufficient in only meat production. Similarly, the West African Economic and Monetary Union and the Caribbean Community are self-sufficient in only two food groups—legumes, nuts and seeds, and starchy staples for the West African Economic and Monetary Union, and fruits and meat for the Caribbean Community. Four economic unions achieve self-sufficiency for five out of seven food groups, but none surpass this. It is worth noting that no union is self-sufficient in vegetables, and only two achieve self-sufficiency for fish and seafood (Fig. 2).

While regional trade enhances self-sufficiency, it also exposes countries to risks if they rely too heavily on a narrow group of trade partners. For example, West Africa’s high dependence on rice imports—70% in some countries—makes the region vulnerable to market shocks, as seen during the COVID-19 pandemic and the 6 day Ever Given Suez Canal blockage in 2021. These examples underscore the importance of diversifying trade relationships to improve resilience^12^.

Countries characterized by low self-sufficiency and high reliance on a single or a few trading partners face increased vulnerability to disruptions. Many countries producing less than half of the vegetables needed domestically, such as those in sub-Saharan Africa and East Asia, rely on a single country for over half of their imports (Supplementary Fig. 3). Similarly, countries in Central America and the Caribbean depend on the United States for starchy staples, while several European and Central Asian countries source most of their legumes, nuts and seeds from a single country. This pattern is especially pronounced in smaller nations, including island states.

Trade between countries with surplus food production and those with shortfalls can notably boost self-sufficiency. Intra-union trade leads to an average improvement in self-sufficiency of 0.27 food groups, with the Republic of Congo, Malaysia, Cambodia, Afghanistan and Malta benefiting most, increasing by three food groups. At a broader, United Nations (UN) world region level, self-sufficiency improves by 1.43 food groups on average, island countries benefitting most.

However, nutrient availability can still be affected by trade dynamics. For instance, the perishable nature of meat and dairy may limit access to essential micronutrients such as calcium and vitamin B_12_ in regions with low self-sufficiency^13^. High self-sufficiency in fruits, as seen Latin America, can result in reduced vitamin C availability potentially linked to substantial exports.

These findings hold true when using other global dietary guidelines, such as the EAT-Lancet diet (Supplementary Fig. 2). In fact, countries perform worse under this model, with 17 countries achieving no food group requirements and none achieving self-sufficiency in more than five food groups, largely due to higher requirements for legumes, nuts and seeds, where only 16% of countries are self-sufficient when applying EAT-Lancet dietary requirements. The numbers of countries able to meet these requirements through domestic production reduces by 24 in Africa, 12 in the Americas and 8 in Asia; only Oceania maintains comparatively high coverage. Self-sufficiency in starchy staples (52%) and fish and seafood (28%) is slightly higher than according to the Livewell diet, while it is also lower in meat (57%), dairy (36%), fruits (36%) and vegetables (16%). Outside of Europe and Asia only eight countries achieve vegetable self-sufficiency.

Current analyses provide a snapshot of production and self-sufficiency, revealing that many countries fall short of their domestic food needs. This does not necessarily indicate an inability to produce sufficiently; cheaper imports and structural constraints (for example, cropping cycles, infrastructure limitations) may suppress production. However, current production volumes serve as a reliable indicator of a country’s capacity to quickly respond to trade disruptions.

In the medium to long term, reallocating resources and investing in technologies could substantially enhance production capacities. Advancements in agriculture and aquaculture, such as genetic engineering, precision farming, controlled environment agriculture and cellular agriculture, and strategies such as Singapore’s ‘30 by 30’ strategy—to sustainably produce 30% of the country’s nutritional needs by 2030—demonstrate the potential for enhancing domestic food production^14^. In analysing the change in self-sufficiency from 2020 to 2032, projections suggest that almost all countries have the potential to improve self-sufficiency, although this varies across different food groups (Supplementary Note 1 and Supplementary Fig. 4).

Projections from 2020 to 2032 show potential for increasing self-sufficiency in animal-source foods, mostly attributed to meat. Countries currently not self-sufficient in meat could close the gap by an average of 12 percentage points. Meat production shows considerable potential for improvement in nine countries, with the Middle East and North Africa expected to reduce the self-sufficiency gap by 28 percentage points, followed by sub-Saharan Africa at 13 percentage points. However, despite this potential, only five countries are projected to achieve full self-sufficiency in dairy production; the overall dairy gap is expected to narrow by just 6 percentage points. Fish and seafood production also shows limited potential for improvement, with the global fish self-sufficiency gap projected to narrow by only 2 percentage points, with only 2 countries reaching full sufficiency. Sub-Saharan Africa could increase fish production by a modest average of 5 percentage points, although other regions such as Latin America and the Caribbean are expected to see declines in production (Supplementary Fig. 4).

For plant foods, projections indicate strong potential for closing self-sufficiency gaps. For legumes, nuts and seeds, countries not self-sufficient could narrow the gap by an average of 19 percentage points, driven largely by gains in Europe, Central Asia and sub-Saharan Africa. Starchy staple production also shows promise, particularly in sub-Saharan Africa, which is projected to close the gap by 15 percentage points. Fruits and vegetables, which—due to lack of data—were not included in the 2032 projections, have shown a 3–4 percentage points increase in self-sufficiency between 2010 and 2020, primarily due to gains in Asia (fruits) and Africa and the Americas (vegetables). (Supplementary Table 2 and Supplementary Fig. 5).

When accounting for future estimated changes in dietary needs, these projections broadly become more conservative in that countries see smaller gains toward achieving self-sufficiency (Supplementary Fig. 6). Although current projections highlight notable limitations in food self-sufficiency across many regions, it is essential to recognize the dynamic potential for future improvements, which may not be fully captured in these production forecasts.

National policymakers increasingly emphasize the importance of consuming domestically produced food, with growing discussions around independence and self-reliance in various economic sectors. These findings underscore that achieving comprehensive nutritional goals requires international cooperation. This holds true for WWF’s Livewell diet as well as the other global dietary guidelines, such as the EAT-Lancet diet. Many countries, particularly in the Caribbean, West Africa and Gulf states, depend heavily on trade to meet their food needs. Although food trade has both human^15^ and environmental costs^16^, transport contributes relatively little to food system emissions^7,16^. Trade plays a crucial role in diversifying food supply and enhancing resilience to market shocks^17^. However, an overdependence on a limited number of trade partners leaves countries vulnerable to disruptions, underscoring the importance of building a diversified trade portfolio to maintain high response diversity^12^.

As dietary needs rise with population growth, sub-Saharan Africa may struggle to meet demand through own production growth, while countries in Europe are projected to achieve greater self-sufficiency in legumes, nuts and seeds. At the same time, the overproduction of resource-intensive animal-sourced foods in many regions necessitates a systematic shift in consumption and production patterns, supported by public policies that promote sustainable diets.

While current data and projections provide valuable insights into future self-sufficiency, they do not account for potential sectoral shifts driven by policy changes or economic incentives. Countries may alter their production focus in response to future trade policies, technological advances or other changes. Future research should develop dynamic models that better capture these factors and incorporate more granular trade dependencies, as they could markedly reshape the self-sufficiency outlook.

## Methods

### Data sources

We used four main data sources for our analysis: (1) FAO FBS, (2) agricultural projections from the Organisation for Economic Cooperation and Development (OECD)-FAO Agricultural Outlook 2023–2032, (3) age-specific food group intake recommendations for individuals and (4) the UN World Population Prospects. We utilize production data sourced from the FAO FBS for the years 2010 to 2022. Our main analysis uses data from 2020, the most recent year available when we started with the analysis. We adjust the production data by food used for feed, food that is lost throughout the food-value chain, utilization for non-food purposes and allocation for seeding, all provided by the FAO FBS. In addition, we use data provided by Gustavsson et al.^18^ to consider the fraction of nonedible and wasted food. We divide the estimated national food supply by the population size in 2020 to obtain per capita daily food supply in grams.

We use projections for the year 2032 for food items of five food groups, namely, starchy staples; meat; dairy products; fish and seafood; and legumes, nuts and seeds from the OECD-FAO Agricultural Outlook 2023–2032. These projections are based on the Agricultural Linkage–Commodity Simulation Model, a recursive-dynamic, partial equilibrium model designed to simulate annual market balances and price developments for major agricultural commodities produced, consumed and traded globally. It links country-level agricultural production data to global commodity market forecasts using data from the FAO and OECD. This model was chosen due to its ability to incorporate a wide range of factors influencing production, including land-use changes, technology adoption, trade and policy shifts. The model works under the assumption that existing trade policies remain unchanged, while considering trade agreements ratified until the end of 2022. Projections are available for 33 populous non-European Union countries and all European Union member states as well as for aggregate regions. However, it is important to note that no projections are available for fruits and vegetables. This limitation may impact the overall conclusions, particularly for regions where fruits and vegetables contribute considerably to the diet and trade dynamics. For countries without data, we use regional projections. No projections are available for ‘other meat’ and ‘offals’, where we use country-specific mean growth rates of the four remaining meat food items.

We use trade data from the FAO Food and Agricultural Trade Dataset, which follows the standard International Merchandise Trade Statistics methodology. This dataset primarily relies on data provided by the UN Statistics Division, Eurostat and other national authorities. However, it does not include data on aquaculture trade, so this food group is excluded from our trade dependency analysis. We analyse trade volumes of individual food items, grouping them into categories consistent with the analyses using the FAO FBS. This allows us to determine the proportion of imports originating from the leading supplier country, offering insights into response diversity—the ability of countries to adapt to trade disruptions by diversifying their import sources. Response diversity was calculated by measuring the proportion of a country’s imports from a single supplier. Low diversity indicates higher vulnerability to trade shocks. However, aquaculture trade is excluded from this analysis due to insufficient data. For our recommendations for seven food groups, we draw upon the WWF’s ‘Livewell diet’. This diet gives per capita, per-day recommendations for 29 specific food items, tailored to four distinct age groups, designed to promote both health and sustainability (WWF Technical Report). The diet is constructed optimizing the composition of current diets by applying (a) nutritional constraints based on the Eatwell Guide recommendations (such as eating at least five portions of fruits and vegetables per day); (b) environmental constraints regarding CO_2_ emissions, water use, eutrophication and biodiversity loss from the Harmonised Environmental Storage and Tracking of the Impacts of Agriculture database; (c) costs of diets; and (d) an acceptable and realistic scenario by limiting dietary changes within a realistic range (for details, see ref. ^9^). Nutritional constraints are based on age-specific dietary reference values that estimate energy and nutrient requirements for healthy populations. The diet is constructed using an optimization tool employing quadratic programming to optimize the composition of current diets to concurrently enhance nutritional and environmental outcomes while keeping current energy intake constant. The nuanced decomposition of this diet enables us to take variations in the demographic composition of countries into account which, in this case, represents an advantage over the EAT-Lancet diet. By tailoring the recommendations to specific age groups and considering demographic shifts, this allows for a more accurate estimation of national food needs. This is particularly important when analysing countries with young or ageing populations, where dietary requirements can vary considerably from the global average. It is important to note that the guidelines in both diets align closely for adults. The specific values used for both diets are provided in Supplementary Table 3.

The UN World Population Prospects provides country-level population estimates, in terms of the total population size as well as the proportion of each country’s population by age. We calculate the estimated population for each age group in 2020 for which dietary recommendations are available.

### Data analysis

To calculate national food supply, we adjust the production data by food used for feed, food that is lost throughout the food-value chain, utilization for non-food purposes, and allocation for seeding, all provided by the FAO FBS. In addition, we use data provided by Gustavsson et al.^18^ to consider the fraction of nonedible and wasted food. We divide the estimated national food supply by the population size in 2020 to per capita daily food supply in grams. We impose the restriction that a food item cannot have negative values when adjusted, which is the case when more of an item is used for feed, non-food uses or wasted than domestically produced. In doing so, we avoid making strong assumptions on the substitutability of food items within the same food group; for example, if more soybean is used for feed than produced, overproduction of beans or lentils cannot make up for this. In these cases, the food item will take the value 0. While we account for food used for feed, seeding, nonfood uses, and food lost and wasted, some food items change their food group when being processed, for instance, when making oil from rapeseeds. Therefore, we subtract the amount processed from oilseeds and crops such as sunflower seeds, soyabeans, sesame seeds, olives, other oilcrops and coconuts.

To compute the dietary requirements for various food groups, we start by multiplying the recommended intake for each specific age group by the population of that age group within the country. This calculation is carried out for all age groups, and the results are then summed together to derive the total dietary requirements for the entire population. To obtain per capita dietary needs, we subsequently divide the national dietary requirements by the total population, encompassing individuals of all age groups. This yields the following equation, where *g* is the age group and *c* the country:

$${\rm{need}}_{c}=\frac{{\sum}_{g=1}^{N}{\rm{recommendation}}_{c,g}\times {\rm{population}}_{c,g}}{{\rm{Total}}\,{\rm{population}}_{c}}$$

The gap in national food production is then the difference between the daily intake needs in grams and the per capita supply.

To analyse past trends in self-sufficiency, we use production and population data from the respective years to calculate year- and country-specific dietary requirements and food availability from domestic production. Production data are adjusted by food used for feed, food that is lost throughout the food-value chain, utilization for non-food purposes and allocation for seeding from the respective year. Food wasted at the household level and non-edible parts are taken from Gustavsson et al.^18^. For comparison of 2010 with other points in time, we restrict the sample to countries with available data in 2010. While we apply growth projections about future food item-specific feed use, no projections for food loss and waste, non-food uses other than biofuel and food used for seed are available in this dataset. Therefore, we take the values from 2020.

### Reporting summary

Further information on research design is available in the Nature Portfolio Reporting Summary linked to this article.

## Supplementary information

Supplementary Information Supplementary Figs. 1–6, Tables 1–3 and Note 1.

Reporting Summary

## Data Availability

All data are publicly available. Food production data from the FAO FBS are available via the Food and Agriculture Organization Statistics (FAOSTAT) at https://www.fao.org/faostat/en/#data/FBS (accessed on 24 July 2024). Food trade data are available at FAOSTAT at https://www.fao.org/faostat/en/#data/TM (accessed on 18 June 2024). Population data are available through the UN’s population division at https://population.un.org/wpp/ (accessed on 23 July 2024). OECD–FAO Agricultural Outlook 2023–2032 data are available via the OECD’s Data Explorer (accessed on 1 July 2024). Livewell food group recommended intake levels are available through the World Wildlife Fund’s 2023 ‘Eating for Net Zero’ technical report^9^ (accessed on 7 June 2024). Food waste and edible portions are available in Gustavsson et al.^18^.

## Code availability

All STATA code used for analysis in this paper is available with open access via GitHub at https://github.com/JonasStl/SelfSufficiency.git.
