# Supplementary material for: Gap between national food production and food-based dietary guidance highlights lack of national self-sufficiency
Source: Nat Food. 2025 May 16;6(6):571–6. doi: 10.1038/s43016-025-01173-4 (PMC12185324; doi:10.1038/s43016-025-01173-4)
Supplement: Supplementary file 1 — Supplementary Figs. 1–6, Tables 1–3 and Note 1. [file 43016_2025_1173_MOESM1_ESM.pdf]

nature food

Supplementary information https://doi.org/10.1038/s43016-025-01173-4

Gap between national food production and food-based dietary guidance highlights lack of national self-sufficiency

In the format provided by the authors and unedited

Supplementary Figures

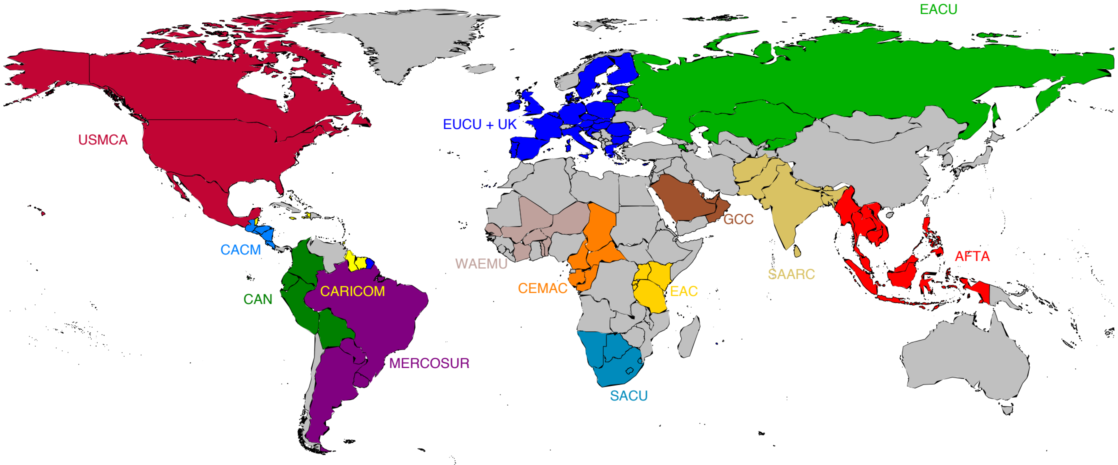

Supplementary Figure 1. World map of economic unions used in this analysis.

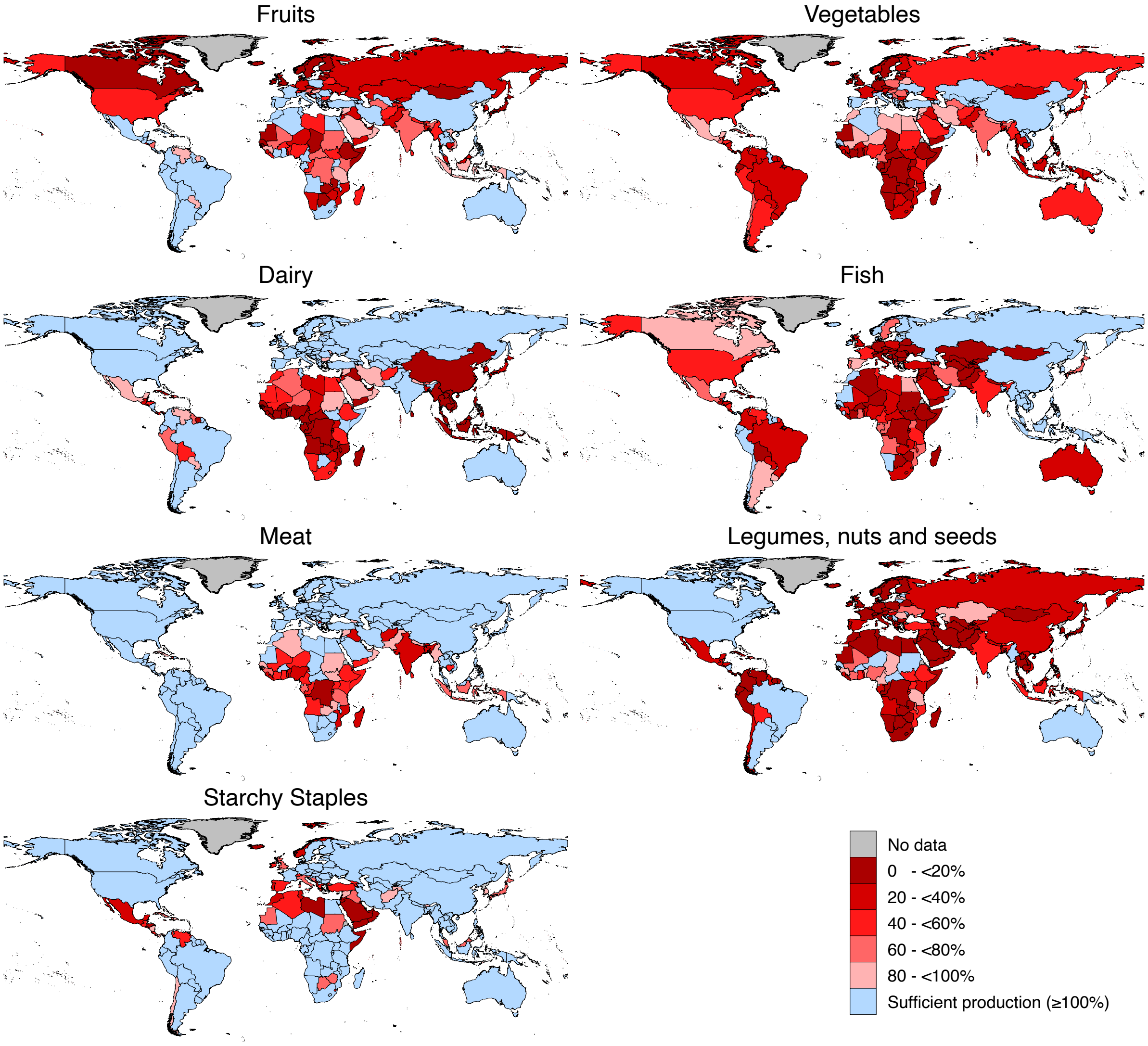

Supplementary Figure 2. Percentage of self-sufficiency for specific food groups according to EAT-Lancet. This figure shows national food availability from domestic production as proportion from recommended intake by the EAT-Lancet diet in grams per capita per day for 187 (dairy: 186) countries in 2020.

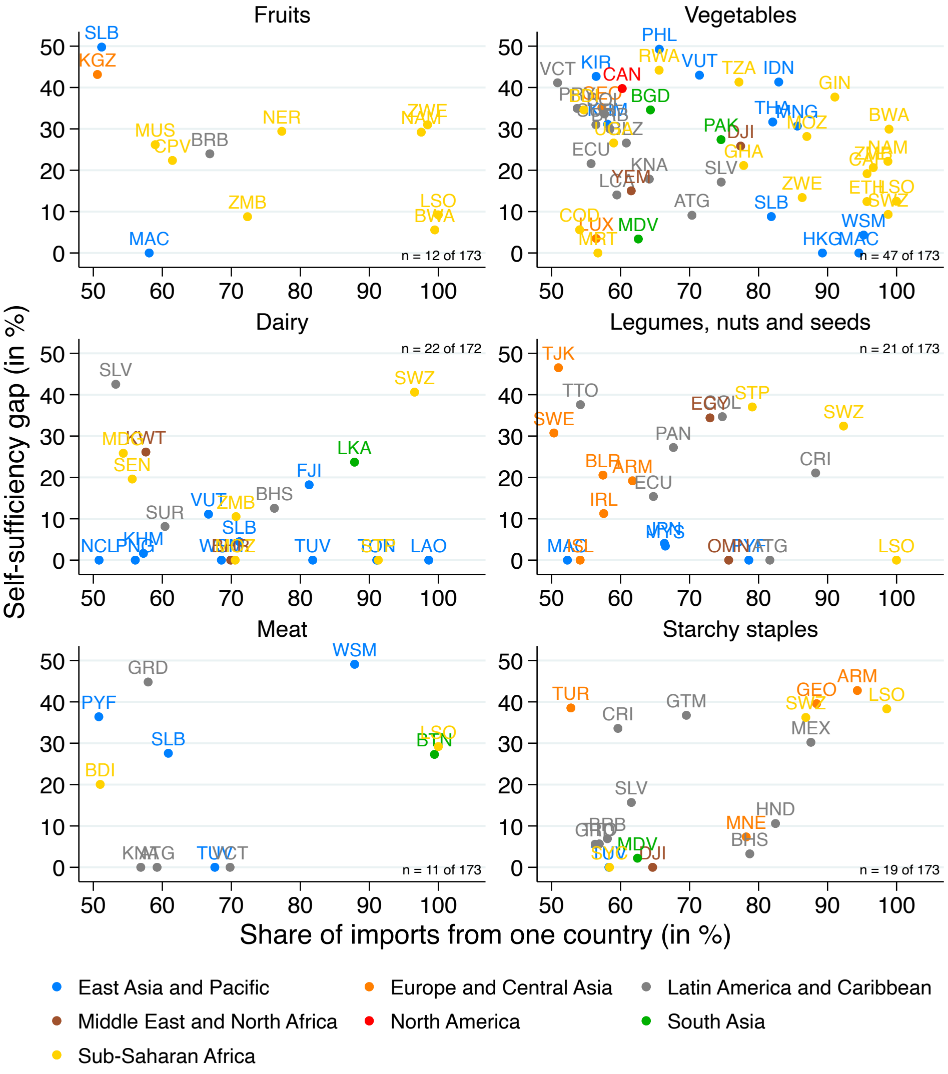

Supplementary Figure 3. Self-sufficiency and response diversity. This graph shows countries with less than 50 percent self-sufficiency that import more than 50 percent of the respective food group from one country for six food group with available trade data. The number at the bottom right indicates the total number of countries fulfilling these criteria and the total sample considered.

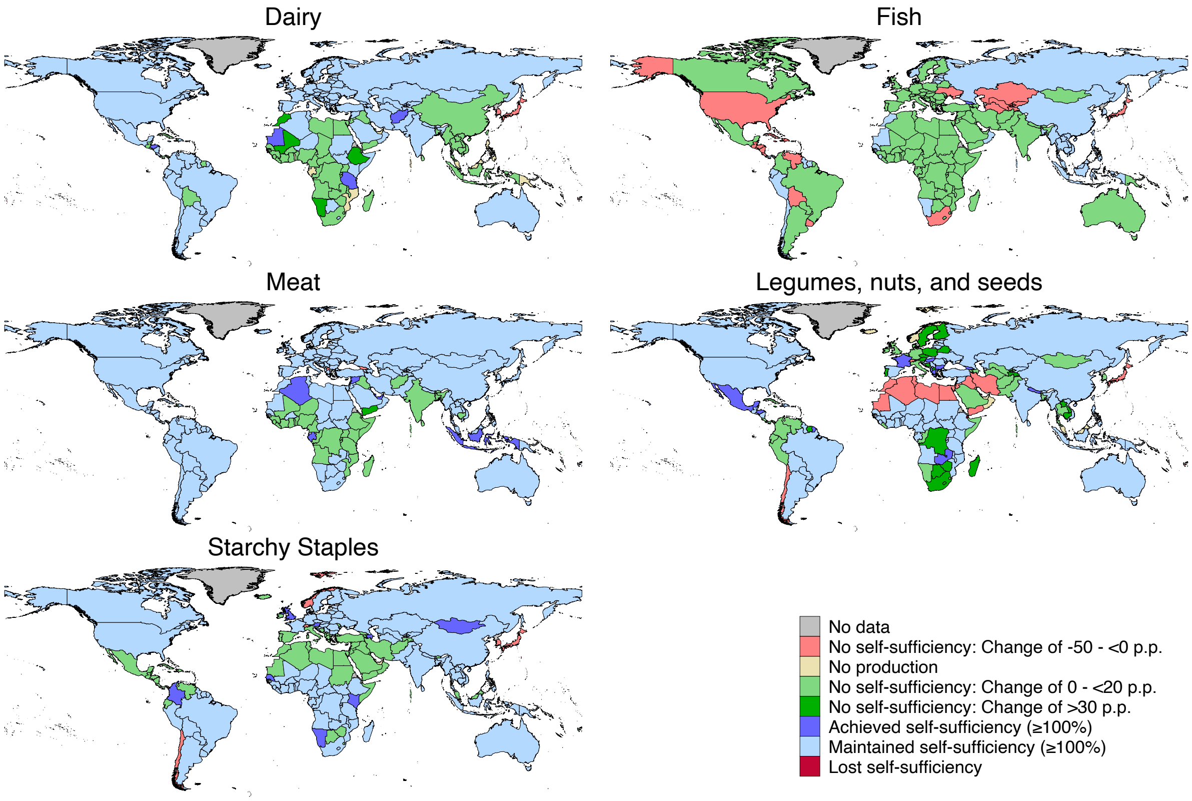

Supplementary Figure 4. Change in self-sufficiency with forecasted 2032 production. This figure depicts changes in national food self-sufficiency using forecasted changes in production for 2032 production of five food groups. Recommended intake is used from the Livewell diet. Red and green show changes in self-sufficiency in percentage points for countries that produce insufficiently for domestic needs using both, production in 2020 and production in 2032 as a proxy for production capacities. Countries in blue have maintained self-sufficiency (light blue) or achieved self-sufficiency (dark blue) with forecasted 2032 production.

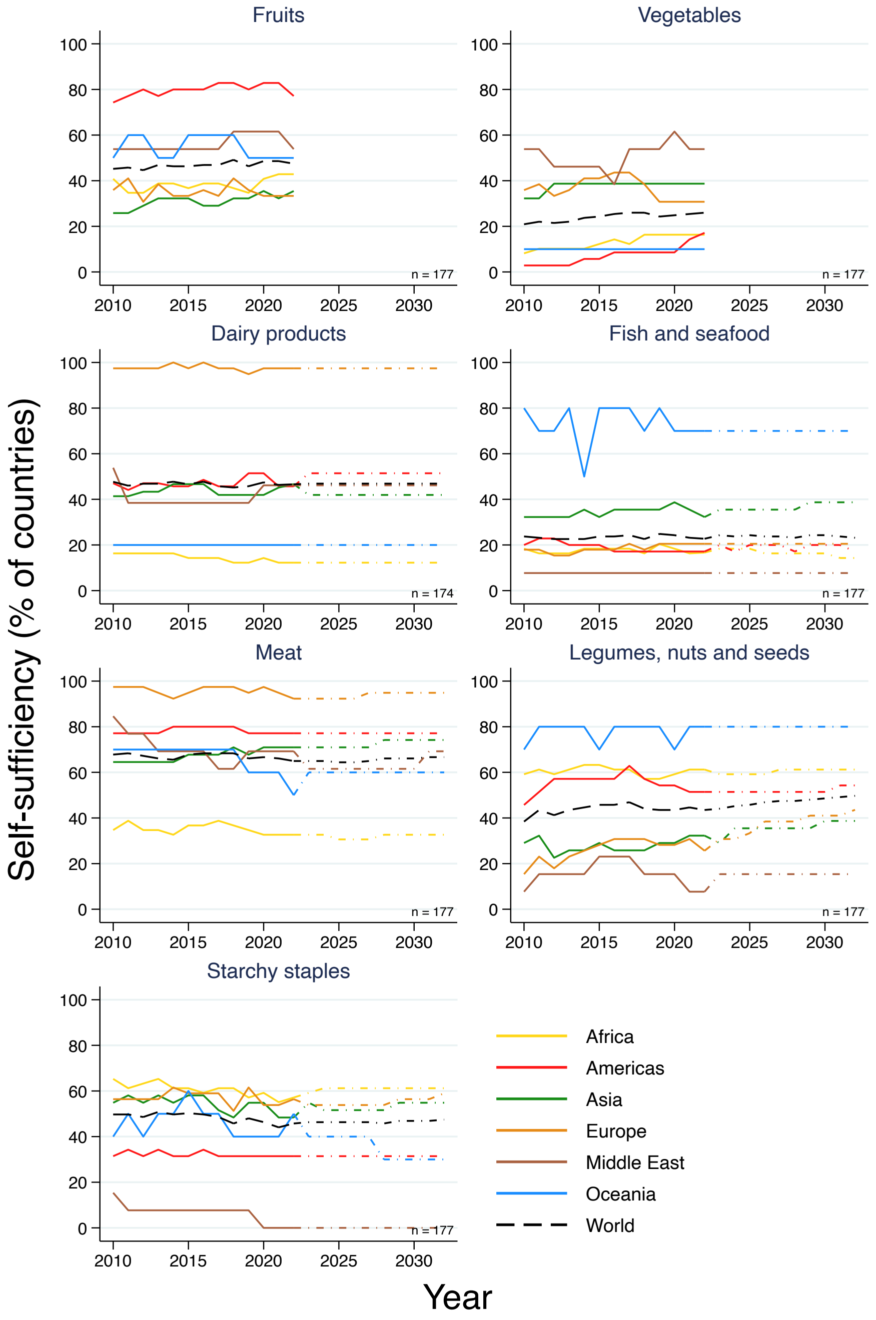

Supplementary Figure 5. Trends in self-sufficiency. This graph depicts the proportion of self-sufficient countries for each world region and the world for fruits and vegetables between 2010 and 2022 and all other food groups between 2010 and 2032. All values between 2010 and 2022 are based on the FAO Food Balance Sheets. All values between 2023 and 2032 are based on food item-specific production growth rates from the OECD-FAO Agricultural Outlook.

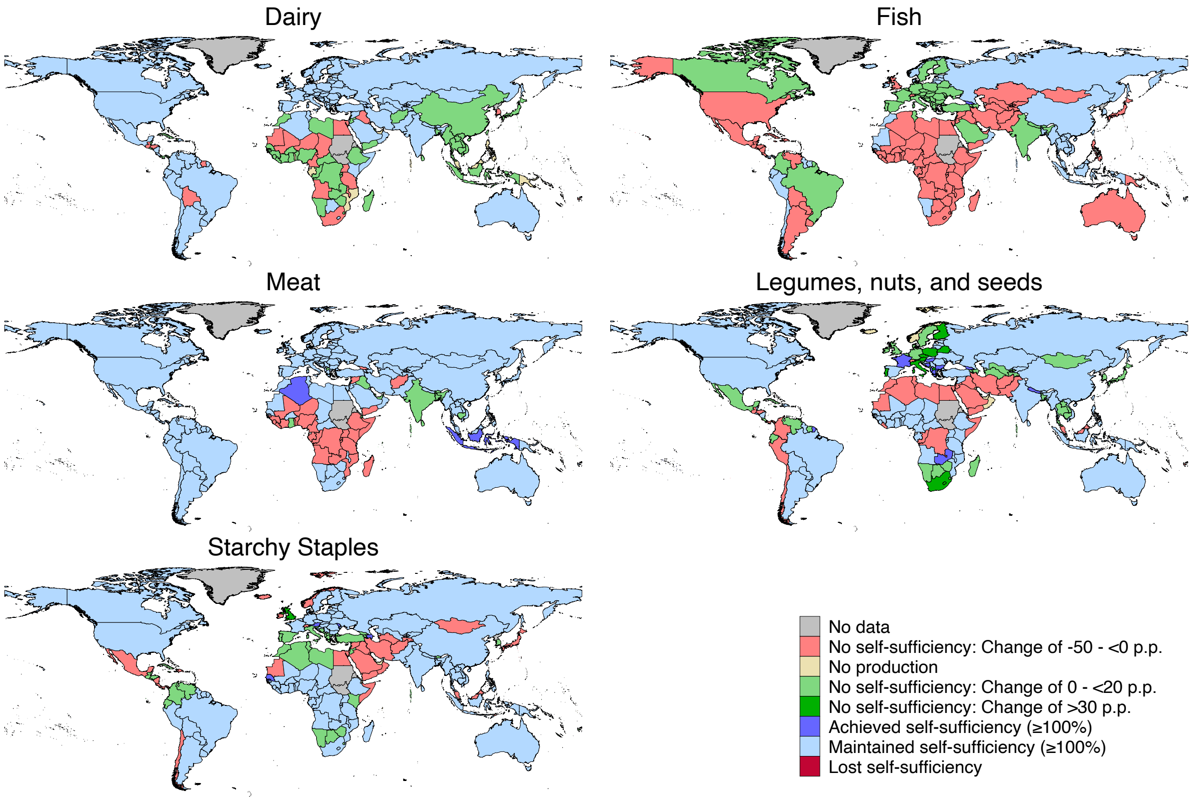

Supplementary Figure 6. Expected changes in self-sufficiency for 2032. This figure depicts changes in national food self-sufficiency using both, expected changes in dietary needs and forecasted changes in production for 2032 of five food groups. Recommended intake is used from the Livewell diet. Red and green show changes in self-sufficiency in percentage points between 2020 and 2032 for countries that produce insufficiently for domestic needs in both years. Countries in blue have maintained self-sufficiency (light blue) or achieved self-sufficiency (dark blue) in 2032.

Supplementary Tables

Supplementary Table 1. Assignment of countries to economic unions.

ASEAN Free Trade Area (**AFTA**)	Brunei, Cambodia, Indonesia, Lao PDR, Malaysia, Myanmar, Philippines, Singapore, Thailand, Vietnam
Central American Common Market (**CACM**)	Costa Rica, El Salvador, Guatemala, Honduras, Nicaragua
Andean Community (**CAN**)	Bolivia, Colombia, Ecuador, Peru
Caribbean Community (**CARICOM**)	Antigua and Barbuda, The Bahamas, Barbados, Belize, Dominica, Grenada, Guyana, Haiti, Jamaica, St. Kitts and Nevis, St. Lucia, St. Vincent and the Grenadines, Suriname, Trinidad and Tobago
Communauté Économique et Monétaire de l'Afrique Centrale (**CEMAC**)	Cameroon, Central African Republic, Chad, Rep. Congo, Gabon
East African Community (**EAC**)	Burundi, Kenya, Rwanda, Tanzania, Uganda
Eurasian Customs Union (**EACU**)	Armenia, Belarus, Kazakhstan, Kyrgyz Republic, Russian Federation
European Union Customs Union and United Kingdom (**EUCU+UK**)	Austria, Belgium, Bulgaria, Croatia, Cyprus, Czech Republic, Denmark, Estonia, Finland, France, Germany, Greece, Hungary, Ireland, Italy, Latvia, Lithuania, Luxembourg, Malta, Netherlands, Poland, Portugal, Romania, Slovak Republic, Slovenia, Spain, Sweden, United Kingdom
Gulf Cooperation Council (**GCC**)	Bahrain, Kuwait, Oman, Qatar, Saudi Arabia, United Arab Emirates
**MERCOSUR**	Argentina, Brazil, Paraguay, Uruguay
South Asian Association for Regional Cooperation (**SAARC**)	Afghanistan, Bangladesh, Bhutan, India, Maldives, Nepal, Pakistan, Sri Lanka
Southern African Customs Union (**SACU**)	Botswana, Eswatini, Lesotho, Namibia, South Africa
United States-Mexico-Canada Agreement (**USMCA**)	Canada, Mexico, United States
West African Economic and Monetary Union (**WAEMU**)	Benin, Burkina Faso, Côte d'Ivoire, Mali, Niger, Senegal, Togo

Supplementary Table 2. Trends in self-sufficiency for three points in time: 2010, 2020 and 2032. Panel 1 shows the proportion of countries self-sufficient in three points in time. Panel 2 shows the average share of self-sufficiency of countries not self-sufficient in 2022, e.g. with less than 100% of their dietary needs met with domestic production in 2022.

	(1) Fruits	(2) Vegetables	(3) Dairy products	(4) Fish and seafood	(5) Meat	(6) Legumes/ nuts/seeds	(7) Starchy Staples	Avg. # of food groups self-sufficient: Of 7: (1)-(7)	Of 5: (3)-(7)
Panel 1: % of countries self-sufficient									
2010	45.20	20.90	47.70	23.73	67.80	38.42	49.72	2.96	2.29
2020	48.59	24.86	47.46	24.29	66.67	43.50	46.33	3.06	2.31
2032			46.89	23.16	66.67	49.72	47.46		2.37
Panel 2: Self-sufficiency of countries <100% in 2022									
2010	46.11	37.96	30.15	26.77	54.32	37.86	43.02		
2020	42.40	43.15	27.83	27.91	57.70	67.46	41.40		
2032			29.57	27.01	58.74	97.78	43.60		
Sample	177	177	174	177	177	177	177	174	174

Supplementary Table 3. Livewell food group consumption recommendations by age group in the World Wildlife Fund's 2023 technical report and EAT-Lancet food group recommendations. All values reported as grams/day. Food group categories aggregated from the WWF Livewell food categories: total meats = 'beef', 'lamb', 'pork', 'offal', 'poultry', and 'processed red meat'; fruits = 'fruit'; legumes nuts seeds = 'legumes, nuts and oilseeds'; dairy = 'milk and milk products', 'cheese'; fish seafood = 'white fish', 'oily fish', 'shellfish'; starchy staples = 'cereal and other cereal products', 'potatoes'; vegetables = 'vegetables' (does not include potatoes or legumes).

Food group	Livewell diet					EAT-Lancet diet
	19-64y	1.5-3y	4-10y	11-18y	65+y	
Total meats	36.51	22.52	44.14	30.85	31.11	43
Fruits	158.58	136.21	145.03	106.89	150.37	200
Legumes/nuts/seeds	36.40	14.81	18.10	20.69	22.02	125
Dairy	147.32	297.81	109.47	189.51	210.60	250
Fish seafood	40.84	14.04	18.73	26.77	40.71	28
Starchy staples	390.60	191.35	263.75	396.65	310.59	282
Vegetables	265.81	110.18	154.97	248.32	203.73	300

Supplementary Notes

Supplementary Note 1: Production capabilities

Self-sufficiency provides insights into current production patterns, not longer-term production capabilities

Our main analysis provides a snapshot of current self-sufficiency patterns, defining self-sufficiency as the ability to produce enough domestically to meet national dietary requirements. While countries typically import a portion of their domestic consumption, it is likely that they could increase production if needed or if circumstances forced them to do so. This potential hinges on agricultural input factors such as crop yields, cropping intensity, land use in the crop sector, and the number of farm animals and output per animal in the livestock sector.

Some of these input factors can be increased, though primarily in the medium to long term. Countries could redirect land, labor, and capital toward the production of specific commodities. However, in the crop sector, benefiting from these adjustments would require at least one more agricultural cycle, which may be too slow to mitigate immediate global trade or price shocks. Moreover, focusing on a single crop or livestock product is neither desirable nor realistic, as it would severely limit the domestic production of other foods.

To maintain a realistic approach, we rely on agricultural growth projections from the OECD-FAO Agricultural Outlook Database. These projections help us assess current capabilities and forecast future self-sufficiency, without assuming theoretical maximum production[20]. These forecasts aim to provide a realistic outlook on the future availability and use of agricultural land, technological improvements, cropping intensity, and considerations of environmental and animal welfare

regulations. A more detailed discussion of agricultural growth forecasts and countries' capabilities and limitations follows below.

Expected growth in agricultural production

Key factors driving agricultural production include crop yields, cropping intensity, and land use in the crop sector, alongside the number of farm animals and their output in the livestock sector. Over the next decade (2023-2032), global production growth is expected to decelerate to an annual rate of 1.1%, primarily due to rising costs—particularly for energy and fertilizers—and the impact of stricter environmental regulations, compounded by lower gross returns from sales[21(p42)].

This production growth will be almost entirely driven by resource-constrained regions such as China, India, and other Asian countries, where productivity gains—largely through increased yields—will be the main driver[21(p42)]. These gains are anticipated to result from investments in agricultural infrastructure, research and development, the mobilization of critical resources like land and irrigation water, more intensive use of agricultural inputs, and improved management practices. By 2032, Asia is projected to account for roughly half of the world's crop and livestock production and nearly three-quarters of global fish production[21(p42)].

In Sub-Saharan Africa and the Middle East and North Africa, significant production growth is expected, albeit from a relatively low baseline. While crop production currently dominates agricultural output in these regions, livestock production is forecasted to outpace crops over the next decade, driven by rapid population growth and urbanization[21(p43)].

Sub-Saharan Africa's crop production growth will be spurred by a combination of area expansion, changes in crop mix, and notable productivity improvements, with yields expected to double compared to the previous decade. This growth is underpinned by investments in improved crop varieties, better management practices, and the expansion and intensification of poultry flocks[21(p44)]. The region's livestock production growth will also be significantly influenced by the dairy sector.

In the Middle East and North Africa, productivity gains will be the primary driver for crop production growth, given the region's severe limitations in arable land and water availability [21(p24)]. Livestock production increases will be predominantly driven by the expansion of poultry farming.

Europe and Central Asia are projected to see the slowest production growth, with the exception of resource-constrained areas like Eastern Europe and Central Asia. As agricultural land use continues to decline, growth will largely hinge on productivity improvements. However, these gains are likely to be moderated by stringent animal welfare and environmental sustainability regulations[21(p44)].

Similarly, in North America, production growth will be primarily driven by productivity gains, with more substantial growth expected in the crop sector compared to the livestock sector[21(p44)].

In Latin America and the Caribbean, production growth is projected to slow compared to the previous decade, with much of this growth stemming from increased crop yields[20(p44)]. The region's abundance of land will continue to support strong crop production, driven by both

expansion and intensification. However, yield improvements are expected to play an increasingly significant role, driven by a rapid rise in fertilizer use.
