# Supplementary material for: Gap between national food production and food-based dietary guidance highlights lack of national self-sufficiency
Source: Nat Food. 2025 May 16;6(6):571–6. doi: 10.1038/s43016-025-01173-4 (PMC12185324; doi:10.1038/s43016-025-01173-4)
Supplement: Supplementary file 2 — Reporting Summary [file 43016_2025_1173_MOESM2_ESM.pdf]

nature portfolio

Corresponding author(s): Jonas Stehl

Last updated by author(s): Apr 7, 2025

Reporting Summary

Nature Portfolio wishes to improve the reproducibility of the work that we publish. This form provides structure for consistency and transparency in reporting. For further information on Nature Portfolio policies, see our Editorial Policies and the Editorial Policy Checklist.

Statistics

For all statistical analyses, confirm that the following items are present in the figure legend, table legend, main text, or Methods section.

n/a	Confirmed	
☐	☒	The exact sample size (*n*) for each experimental group/condition, given as a discrete number and unit of measurement
☐	☒	A statement on whether measurements were taken from distinct samples or whether the same sample was measured repeatedly
☒	☐	The statistical test(s) used AND whether they are one- or two-sided<br>*Only common tests should be described solely by name; describe more complex techniques in the Methods section.*
☒	☐	A description of all covariates tested
☒	☐	A description of any assumptions or corrections, such as tests of normality and adjustment for multiple comparisons
☒	☐	A full description of the statistical parameters including central tendency (e.g. means) or other basic estimates (e.g. regression coefficient) AND variation (e.g. standard deviation) or associated estimates of uncertainty (e.g. confidence intervals)
☒	☐	For null hypothesis testing, the test statistic (e.g. *F*, *t*, *r*) with confidence intervals, effect sizes, degrees of freedom and *P* value noted<br>*Give P values as exact values whenever suitable.*
☒	☐	For Bayesian analysis, information on the choice of priors and Markov chain Monte Carlo settings
☒	☐	For hierarchical and complex designs, identification of the appropriate level for tests and full reporting of outcomes
☒	☐	Estimates of effect sizes (e.g. Cohen's *d*, Pearson's *r*), indicating how they were calculated

Our web collection on statistics for biologists contains articles on many of the points above.

Software and code

Policy information about availability of computer code

Data collection	This study uses agricultural production data including data on feed, food loss, utilization for non-food purposes, and allocation for seeding from the FAO Food-Balance Sheets. Trade data is sourced from the FAO. Projected future production is taken from the OECD-FAO Agricultural Outlook. Data on food waste and edible portions is used from an FAO report. Population data is used from the UN's population division. Dietary guidance data is used from the WWF's Easting for Net Zero report and the EAT-Lancet commission report.
Data analysis	STATA 17 was used for all analyses. The code is publicly available via GitHub: https://github.com/JonasStl/SelfSufficiency.git

For manuscripts utilizing custom algorithms or software that are central to the research but not yet described in published literature, software must be made available to editors and reviewers. We strongly encourage code deposition in a community repository (e.g. GitHub). See the Nature Portfolio guidelines for submitting code & software for further information.

Data

Policy information about availability of data

All manuscripts must include a data availability statement. This statement should provide the following information, where applicable:

- Accession codes, unique identifiers, or web links for publicly available datasets
- A description of any restrictions on data availability
- For clinical datasets or third party data, please ensure that the statement adheres to our policy

All data is publicly available:

- FAO FBS data are open access through FAOSTAT: https://www.fao.org/faostat/en/#data/FBS.
- Trade Data are open access through FAOSTAT: https://www.fao.org/faostat/en/#data/TM
- OECD-FAO Agricultural Outlook 2023-2032 Data is open access through https://data-explorer.oecd.org/vis?lc=en&df[ds]=dsDisseminateFinalDMZ&df[id]=DSD_AGR%40DF_OUTLOOK_2023_2032&df[ag]=OECD.TAD.ATM&dq=OECD.A.CPC_0111...&pd=2010%2C2032&to[TIME_PERIOD]=false
- UN world population estimates are available through the UN's population division: https://population.un.org/wpp/.
- Livewell food group recommended intake levels are available through the World Wildlife Fund's 2023 'Eating for Net Zero' technical report: https://www.wwf.org.uk/sites/default/files/2023-05/Eating_For_Net_Zero_Technical_Report.pdf.
- EAT-Lancet food group recommended intake levels are available through the EAT-Lancet Summary Report: https://eatforum.org/content/uploads/2019/07/EAT-Lancet_Commission_Summary_Report.pdf
- Food waste at the household and edible portions:
https://www.fao.org/3/i2697e/i2697e.pdf

Human research participants

Policy information about studies involving human research participants and Sex and Gender in Research.

Reporting on sex and gender	Not applicable.
Population characteristics	Age-specific dietary recommendations are included in the analysis.
Recruitment	Not applicable.
Ethics oversight	Not applicable.

Note that full information on the approval of the study protocol must also be provided in the manuscript.

Field-specific reporting

Please select the one below that is the best fit for your research. If you are not sure, read the appropriate sections before making your selection.

☐ Life sciences ☒ Behavioural & social sciences ☐ Ecological, evolutionary & environmental sciences

For a reference copy of the document with all sections, see nature.com/documents/nr-reporting-summary-flat.pdf

Behavioural & social sciences study design

All studies must disclose on these points even when the disclosure is negative.

Study description	Quantitative analysis of the gap between national food production and food-based dietary guidance on country- and regional-levels. Production data was adjusted for food used for feed, food loss, utilization for non-food purposes, allocation for seeding, food waste and edible portions. Food needs were calculated using food group recommended intake levels and population data.
Research sample	Food production data for each country and year was obtained from the FAO Food Balance Sheets (accessed on July 24, 2024). Bilateral food trade data were obtained from FAOSTAT's Trade Matrix (accessed on June 18, 2024). Population data are sourced from the UN's Population Division (accessed on July 23, 2024). OECD-FAO Agricultural Outlook 2023-2032 data are sourced from the OECD's Data Explorer (accessed on July 1, 2024). Livewell food group recommended intake levels are obtained from the World Wildlife Fund's 2023 'Eating for Net Zero' technical report. Food waste and edible portions are available at Gustavsson et al. (2011).
Sampling strategy	The main sample consists of 187 countries for which production and population data were available. Dairy production data was available for 186 countries. For time trends, we analyzed countries with available data in 2010 to ensure comparability.
Data collection	All data were electronically extracted from the aforementioned databases and reports.
Timing	The data were obtained between 06/24 and 07/24.
Data exclusions	No countries with available data were excluded from the analysis.
Non-participation	No participants/human subjects were involved in the study.
Randomization	Randomization is not applicable to our study because we are using cross-sectional macro data. In our data, one data point reflects a specific country in a given year and therefore do not require a randomization to adequately reflect specific population groups.

Reporting for specific materials, systems and methods

We require information from authors about some types of materials, experimental systems and methods used in many studies. Here, indicate whether each material, system or method listed is relevant to your study. If you are not sure if a list item applies to your research, read the appropriate section before selecting a response.

Materials & experimental systems

n/a	Involved in the study
☒	☐ Antibodies
☒	☐ Eukaryotic cell lines
☒	☐ Palaeontology and archaeology
☒	☐ Animals and other organisms
☒	☐ Clinical data
☒	☐ Dual use research of concern

Methods

n/a	Involved in the study
☒	☐ ChIP-seq
☒	☐ Flow cytometry
☒	☐ MRI-based neuroimaging
